# Return of the Righting Reflex Does Not Portend Recovery of Cognitive Function in Anesthetized Rats

**DOI:** 10.3389/fnsys.2021.762096

**Published:** 2021-11-18

**Authors:** Kathleen F. Vincent, Edlyn R. Zhang, Risako Kato, Angel Cho, Olivia A. Moody, Ken Solt

**Affiliations:** ^1^Anesthesia, Critical Care, and Pain Medicine, Massachusetts’s General Hospital, Boston, MA, United States; ^2^Harvard Medical School, Boston, MA, United States; ^3^Touro College of Osteopathic Medicine, New York, NY, United States

**Keywords:** 5-choice serial reaction time task, anesthesia, consciousness, emergence, cognitive recovery, return of righting reflex

## Abstract

As the number of individuals undergoing general anesthesia rises globally, it becomes increasingly important to understand how consciousness and cognition are restored after anesthesia. In rodents, levels of consciousness are traditionally captured by physiological responses such as the return of righting reflex (RORR). However, tracking the recovery of cognitive function is comparatively difficult. Here we use an operant conditioning task, the 5-choice serial reaction time task (5-CSRTT), to measure sustained attention, working memory, and inhibitory control in male and female rats as they recover from the effects of several different clinical anesthetics. In the 5-CSRTT, rats learn to attend to a five-windowed touchscreen for the presentation of a stimulus. Rats are rewarded with food pellets for selecting the correct window within the time limit. During each session we tracked both the proportion of correct (accuracy) and missed (omissions) responses over time. Cognitive recovery trajectories were assessed after isoflurane (2% for 1 h), sevoflurane (3% for 20 min), propofol (10 mg/kg I.V. bolus), ketamine (50 mg/kg I.V. infusion over 10 min), and dexmedetomidine (20 and 35 μg/kg I.V. infusions over 10 min) for up to 3 h following RORR. Rats were classified as having recovered accuracy performance when four of their last five responses were correct, and as having recovered low omission performance when they missed one or fewer of their last five trials. Following isoflurane, sevoflurane, and propofol anesthesia, the majority (63–88%) of rats recovered both accuracy and low omission performance within an hour of RORR. Following ketamine, accuracy performance recovers within 2 h in most (63%) rats, but low omission performance recovers in only a minority (32%) of rats within 3 h. Finally, following either high or low doses of dexmedetomidine, few rats (25–32%) recover accuracy performance, and even fewer (0–13%) recover low omission performance within 3 h. Regardless of the anesthetic, RORR latency is not correlated with 5-CSRTT performance, which suggests that recovery of neurocognitive function cannot be inferred from changes in levels of consciousness. These results demonstrate how operant conditioning tasks can be used to assess real-time recovery of neurocognitive function following different anesthetic regimens.

## Introduction

Each year, hundreds of millions of surgical patients undergo general anesthesia (Meara et al., 2015; Weiser et al., 2015). Regardless of the specific anesthetic regimen employed, the goal of general anesthesia is to produce a reversible, drug-induced state characterized by unconsciousness, amnesia, analgesia, and lack of movement in response to pain. Crucially, the restoration of normal cognition following anesthesia is necessary for patients to be safely discharged from the hospital. However, despite the ubiquity of general anesthetics in medical practice, our understanding of how cognition is restored following these pharmacologically induced breaks in consciousness is surprisingly limited. Animal models have become an invaluable resource for exploring these questions. With a growing repertoire of techniques becoming widely available for the investigation of the neural substrates of anesthesia (Melonakos et al., 2020; Reimann and Niendorf, 2020), establishing comprehensive methods of probing consciousness and cognition in such models could help elucidate the neural basis of these processes.

In animal models, consciousness is assessed using a variety of physiological measures. The return of the righting reflex (RORR) and specific electroencephalography (EEG) signatures are two correlates of consciousness that are predominant in the rodent anesthesia literature. RORR is a binary measure assessed by placing a rodent in a supine position and measuring the time it takes for the animal to return to all fours. Its simplicity makes it a favored endpoint in studies investigating anesthetic reversal mechanisms (Solt et al., 2011; Wang et al., 2014; Pal et al., 2018) and anesthetic sensitivity (McCarren et al., 2013). It is worth noting, however, that RORR is intact in decerebrated rats (Woods, 1964) and is dissociable from cortical patterns of wakefulness (Gao and Calderon, 2020). Furthermore, recent evidence suggests that cortical EEG patterns are dissociable from arousal states in rodents (Pal et al., 2020).

While EEG and RORR provide imperfect measures of the levels of consciousness, how they relate to what has been termed the “content of consciousness” (Mashour and Hudetz, 2017) is entirely unclear. The content of consciousness includes the information that is cognitively processed and consciously accessed at a given moment (Dehaene and Changeux, 2011). In humans, this can be assessed through verbal report and by using established domain-specific neurocognitive tasks. A recent study in young, healthy human volunteers revealed variable cognitive recovery rates after general anesthesia depending on the cognitive domain assayed, with executive function being the most rapid to return and attention, working memory, and reaction time recovering more gradually over the course of 2–3 h (Mashour et al., 2021). In contrast to humans, the moment-to-moment cognitive state in rodents following anesthesia has never been assessed. Instead, rodent studies largely rely on learning tasks, specifically spatial learning, in the days and weeks following anesthesia to assess cognitive function over time (Culley et al., 2003, 2004a,2004b; Crosby et al., 2005; Lee et al., 2008; Valentim et al., 2008). While more recent studies have amassed a battery of tests, using both learned (mazes) and natural behaviors (food burrowing, freezing) to assess cognitive function (Peng et al., 2016; Zhong et al., 2020), how these behaviors relate to a human postoperative cognitive assessment is not readily apparent.

To better understand how consciousness and cognition are restored in rats during the early stages of anesthetic emergence, we adapted a touchscreen operant conditioning task analogous to human continuous performance tasks, the 5-choice serial reaction time task (5-CSRTT). The 5-CSRTT is a complex cognitive testing paradigm that captures features of sustained attention, spatial working memory, and inhibitory control (Robbins, 2002). Previous work has demonstrated that rats show no persistent impairments on this task by 24 h following isoflurane anesthesia (Hambrecht-Wiedbusch et al., 2019). In this study, we allowed rats to recover in the testing chamber following the administration of several mechanistically distinct inhalational and intravenous anesthetics and monitored performance in the minutes and hours following emergence. By testing rats that are well-trained in the 5-CSRTT following emergence, our goal is to capture the recovery of cognitive function using a behavioral assessment akin to human testing and establish its relationship to RORR latency.

## Materials and Methods

### Animals

The rats used in this study were maintained and treated in accordance with recommendations in the Guide for the Care and Use of Laboratory Animals, National Institutes of Health, and were approved by the Massachusetts General Hospital Institutional Animal Care and Use Committee. All efforts were made to minimize animal suffering. Reporting of the animal research in this study complies with the ARRIVE guidelines (Kilkenny et al., 2012).

Adult Sprague Dawley rats (Charles River Laboratories, Wilmington, MA, United States) were housed in a temperature-controlled room with a 12:12-h light-dark cycle (lights on at 0700). Rats were housed one or two per cage with water available *ad libitum*. Male and female rats (*N* = 4 each) between the ages of 2 and 6 months were used for the experiments. Food was restricted 5 days a week. During food restricted days, rats were given access to food only during cognitive testing and for 1 h following testing (free-feeding period). Weights were recorded daily before testing to ensure weights did not drop below 80% of free-feeding weight. All rats maintained adequate weight for the duration of the study: female weight ranged from 220 to 375 g, male weight ranged from 353 to 470 g.

### 5-Choice Serial Reaction Time Task Training

**FIGURE 1:**
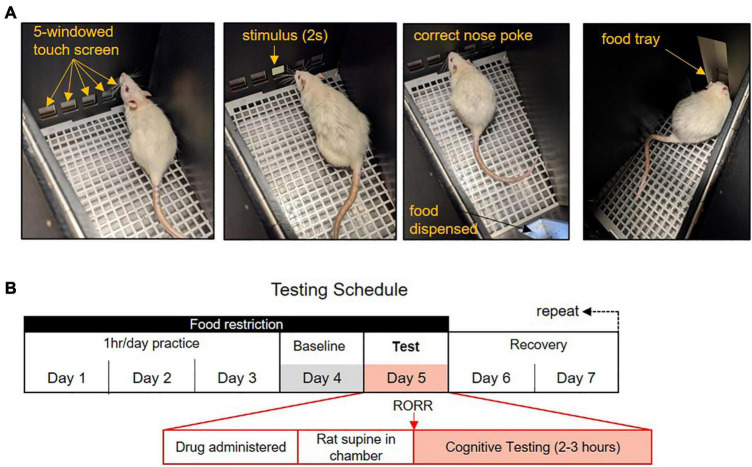
The 5-choice Serial Reaction Time Task (5-CSRTT) testing paradigm. **(A)** In the 5-CSRTT rats attend a five-windowed screen for the presentation of a 2-s stimulus. Rats have 5 s to make a response. Correct responses are rewarded with a food pellet. Trials are initiated with a nose-poke to the food tray. **(B)** Anesthetic testing and food restriction schedule. Rats practice the task 4 days a week for an hour each day. Performance on the fourth day is used as the baseline comparison. On the fifth day, rats are exposed to an anesthetic regimen and placed supine in the testing chamber. Following the return of the righting reflex (RORR), rats may work on the 5-CSRTT for 2–3 h. Rats are given 2 days rest following anesthesia with free food access.

All training was conducted using the Lafayette Instrument Bussey-Saksida Rat Touch Screen Chambers (Model 80604, Lafayette, IN, United States) with the ABETT II Interface and Software packages and the 5-choice Serial Reaction Time Task (5-CSRTT) for Rats (Model 89543-R). The procedure used for the 5-CSRTT training was performed as described in the CAM 5-CSRTT manual. Rats are trained in a trapezoidal room equipped with a touch screen on one side and a magazine food tray on the opposite side (Figure 1A). The touch screen is covered with a windowed mask which restricts screen access to five equal-sized, horizontally spaced, square windows placed at rat eye level. The rat initiates a trial with a nose poke in the magazine and, after a 5 s delay, a white square stimulus appears for 2 s in one of the five windows in a pseudorandom order. The rat’s nose-pokes to the screen are recorded before, during, and after the presentation of the stimulus. Touching the screen before the stimulus appears is recorded as a premature response but is not punished. Touching the correct window within 5 s of the stimulus appearing is recorded as a correct response and the rat is rewarded with the magazine lighting up, the presentation of a tone, and the appearance of a 45 mg grain pellet (Dustless Precision Pellets^®^, Bio-Serv, F0165). There is a 5-s-long inter-trial interval during which no trial can occur to allow the rat to consume the food pellet. The end of the inter-trial interval is indicated by the magazine lighting up. Incorrect responses (touching one of the four windows which did not contain the stimulus) are punished by the illumination of the chamber for 5 s and no food reward. Omissions occur if the rat fails to select a window within 5 s of the stimulus being presented. Omissions are punished by the illumination of the chamber for 5 s and no food reward. Performance on the task is monitored by a live video recording (Gamut 4-channel HD DVR, United Kingdom).

### Analysis of 5-Choice Serial Reaction Time Task Performance

Rats were considered competent at the 5-CSRTT when they could perform > 30 trials in 1 h with > 80% accuracy and < 20% omissions during three consecutive training days. During the task, the following dependent variables were recorded or computed:

Overall accuracy: The percentage of correct responses divided by the total number of both correct and incorrect responses. Rats must achieve > 80% accuracy in > 30 trials in three consecutive days while training to move onto anesthetic testing. Overall accuracy is compared with RORR latency on test days.

Windowed accuracy: Percent accuracy measured using a moving window of five consecutive trials. Only trials in which a response was made are included. When recovering from anesthesia, the time it takes rats to regain > 80% windowed accuracy (i.e., at minimum 4 out of 5 consecutive responses are correct) following RORR is used to measure the recovery of accuracy performance.

Overall omissions: The percentage of the trials in which the rat made no responses divided by the total number of trials completed over time. Rats must achieve < 20% omissions in > 30 trials in three consecutive days while training to move onto anesthetic testing. Overall omissions is compared with RORR latency on test days.

Windowed omissions: Omissions measured in the last five trials the rat initiated. When recovering from anesthesia, the time it takes rats to obtain < 20% moving omissions (i.e., 1 or fewer omissions out of 5 consecutive trials) following RORR is to measure the recovery of omission performance.

Premature responses: The number of times the rat touches the screen during the intertrial interval. This metric is reported as a percent of total trials initiated.

Reward collection latency: The time it takes a rat to enter the food magazine to collect a food pellet after making a correct response.

Correct response latency: The time it takes a rat to touch the correct/incorrect screen after the presentation of the stimulus. Rats have a maximum of 5 s to make a response before the response is recorded as an omission.

Timeout response to correct/incorrect window: The number of responses a rat makes to the correct or incorrect window during a timeout period. Timeouts are initiated following omissions and incorrect responses. The number of timeout responses are reported as a percentage of total trials initiated. Because multiple timeout responses can be recorded during the timeout of a single trial, the percent of timeout responses can exceed 100%.

### Anesthetic Testing

Once competence was achieved, rats trained 4 days a week and were tested under general anesthesia on the fifth day, after which they were given 2 days to recover with free food access and no chamber testing (Figure 1B). Hence, post-anesthetic cognitive testing occurred only once a week. On an anesthetic test day, following administration of the anesthetic drug, rats were placed supine with their nose pointed toward the touch screen in the testing chamber. Return of the righting reflex (RORR), defined as the moment the rat returned to a prone position, was monitored by video surveillance. Rats could complete trials of the 5-CSRTT for 2–3 h following RORR. The amount of food eaten during testing and during the 1-h free-feed period was recorded each day. Performance following anesthesia was compared to the performance of the previous day (baseline). Both training and anesthetic testing occur during the rat’s light cycle, between the hours of 9 a.m. to 3 p.m. The order of anesthetic exposure was held constant for all rats and is as follows: (1) isoflurane, (2) sevoflurane, (3) propofol, (4) high dose of dexmedetomidine, (5) ketamine, (6) low dose dexmedetomidine. The anesthetic regimens are described below.

#### Isoflurane

Anesthesia was induced with 2% isoflurane (Piramal Critical Care Inc., United States) in 100% oxygen and chamber concentration was monitored using the Datex Ohmeda Compact S/5 (Absolute Medical Equipment, New York, United States). The chamber was maintained at 2% isoflurane for 1 h to allow the brain to equilibrate (Lu et al., 2003), at which point rats were removed from the induction chamber and placed supine in the cognitive testing chamber. This dose, which is approximately 1.45–1.55 the minimum alveolar concentration (MAC) (Orliaguet et al., 2001), was chosen as it allows rats to remain unconscious for several minutes following their removal from the induction chamber, permitting them to be placed unconscious in the operant conditioning chambers to recover gradually. RORR was measured as the time taken for the rat to return to a prone position following removal from the induction chamber, captured by video surveillance.

#### Sevoflurane

Anesthesia was induced with 3% sevoflurane (Piramal Critical Care Inc., United States) in 100% oxygen and induction chamber concentration was monitored using the Datex Ohmeda Compact S/5 (Absolute Medical Equipment, New York, United States). The induction chamber was then maintained at 3% sevoflurane for 20 min, allowing the brain to equilibrate (Nakamura et al., 1999). Like isoflurane, this dose, which is estimated to correspond to 1.25–1.4 MAC in adult rats (Orliaguet et al., 2001), was chosen as rats would reliably remain unconscious for several minutes following their removal from the induction chamber. RORR was measured as the time taken for the rat to return to a prone position inside the operant conditioning chamber.

#### Intravenous Anesthetics

A 24-gauge intravenous catheter was placed in the lateral tail vein of each rat in the morning under brief (up to 15 min) isoflurane anesthesia. Rats were given 1 h to recover fully from the tail vein procedure in their home cage prior to test day anesthetic exposure. Propofol (Fresenius Kabi, Austria) was administered as a bolus (10 mg/kg) and flushed with 0.5 mL saline. We have previously found that bolus doses of propofol at 8 mg/kg produces a rapid loss of righting that persists for at least 6 min (Kenny et al., 2015). To provide sufficient time to remove the tail vein catheter, staunch the blood, and allow for conscious recovery in the testing chamber we increased the dose to 10 mg/kg. Dexmedetomidine hydrochloride (AuroMedics Pharma LLC, India) was administered as an infusion over 10 min at both a high (35 μg/kg) and a low (20 μg/kg) dose on separate test days. Dexmedetomidine was administered as an infusion rather than a bolus to avoid transient hypertension, which can result from rapid infusions (Weerink et al., 2017). We have previously observed that 50 μg/kg of dexmedetomidine reliably produces a deeply sedated state with full loss of righting in rats (Kato et al., 2021). However, RORR latency at this dose can exceed 3 h in females. Hence, we initially selected a dose of 35 μg/kg as it was sufficient to induce loss of righting and yielded shorter RORR latencies. However, the profound lack of cognitive recovery prompted the use of a second, lower dose. At 20 μg/kg, rats could be un-righted, but remained responsive to tactile stimuli. Ketamine hydrochloride (Covetrus) was infused over 10 min to a total dose of 50 mg/kg. We have previously observed that infusions at this dose are sufficient to produce a reliable loss of righting in all rats without causing significant respiratory depression (Kato et al., 2021). Immediately following anesthetic infusion, tail vein catheters were quickly removed, the blood was staunched, and the rats were placed supine in the testing chambers. RORR latency was measured as the time it took the rats to return to the prone position inside the testing chamber following the end of the anesthetic administration.

### Statistical Analysis

All data analyses were conducted using GraphPad Prism 6.0. Time to RORR was compared in males and females by two-sample *t*-test. The time to recover moving accuracy and omission performance at baseline and following anesthesia was analyzed by comparing survival curves using the log-rank Mantel-Cox test. Survival curves in this context depicts the probability of a rat recovering task performance at a given time point. This measure was our primary outcome variable of interest. The association between cognitive performance and RORR was assessed for each anesthetic agent using linear regression. The baseline rate of trial initiation between males and females was assessed by linear regression.

Several other performance variables captured by the Lafayette system were also analyzed to provide a broader context of the animal’s performance in the chamber. Premature responses, median reward collection latency, and median correct response latency were compared at baseline and following anesthesia using paired *t*-tests. Normality was tested using D’Agostino and Pearsons omnibus test. When parametric assumptions were violated, as indicated in the results, the appropriate non-parametric tests were employed. Timeout responses to correct and incorrect windows were compared by mixed-factor ANOVA. Dexmedetomidine premature responses and correct trial analyses were performed by Kruskal-Wallis test with Dunn’s multiple comparison test.

The amount of food consumed inside the chamber (measured as 45 mg × the number of correct responses) and the weight of food consumed during the free feeding period at baseline and anesthetic conditions were compared by two-way repeated measures (RM) ANOVA. Except for dexmedetomidine, *post hoc* comparisons were performed using Bonferroni *post hoc* correction for multiple comparisons. Dexmedetomidine *post hoc* testing was performed using Dunnett’s test, which compares both low and high doses of dexmedetomidine to baseline measures.

## Results

### Baseline Performance

**FIGURE 2:**
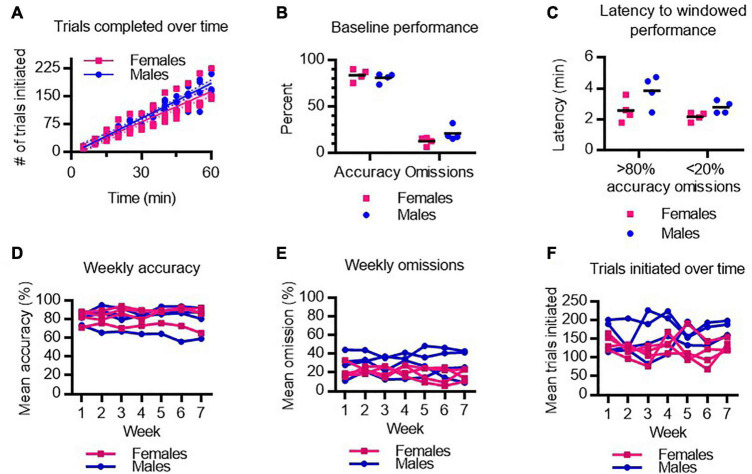
Baseline performance on the 5-CSRTT in male and female rats. **(A)** Number of trials completed by male and female rats following training. **(B)** Average accuracy and omission performance prior to anesthesia testing in male and female rats. **(C)** The latency to achieve 4 out of 5 correct responses (>80% windowed accuracy) and to achieve 1 or fewer omissions out of 5 trials (<20% windowed omissions) at baseline in male and female rats. **(D–F)** Mean accuracy **(D)**, omissions **(E)**, and trials initiated **(F)**, over 7 weeks of testing was stable across male and female rats. *N* = 8.

In the 5-CSRTT, trials are initiated by the rats *ad libitum*. To assess baseline task motivation, the rate at which individual rats initiate trials was compared between male and female rats prior to anesthetic testing (Figure 2A). No sex differences were observed in the rate of trial initiation over the hour-long testing session during baseline performance. A two-way ANOVA revealed no main effect of sex on overall accuracy or omission rates in the 5-CSRTT, indicating male and female rats achieved comparable baseline performance in the 5-CSRTT (Figure 2B). We next assessed how quickly rats achieve > 80% windowed accuracy (≥4 of 5 correct trials in a row) and < 20% windowed omissions (≤1 of 5 trials missed in a row) (Figure 2C). There was no significant difference between males and females in baseline latency to achieve high accuracy and low omission rates. Importantly, the data demonstrate that at baseline, rats rapidly engage in the task with a high degree of competence.

Anesthetic testing occurred over 7 weeks. To determine whether regular practice at the task improved performance over time, or conversely, whether repeated anesthetic testing deteriorated performance over time, we assessed average task performance each week in males and females. We found no main effect of test week on 5-CSRTT performance as measured by accuracy (Figure 2D), omissions (Figure 2E), or number of trials performed (Figure 2F). Hence, task performance is stable and consistent week-to-week following various anesthetic exposures.

### Cognitive Recovery Following Isoflurane Emergence

**FIGURE 3:**
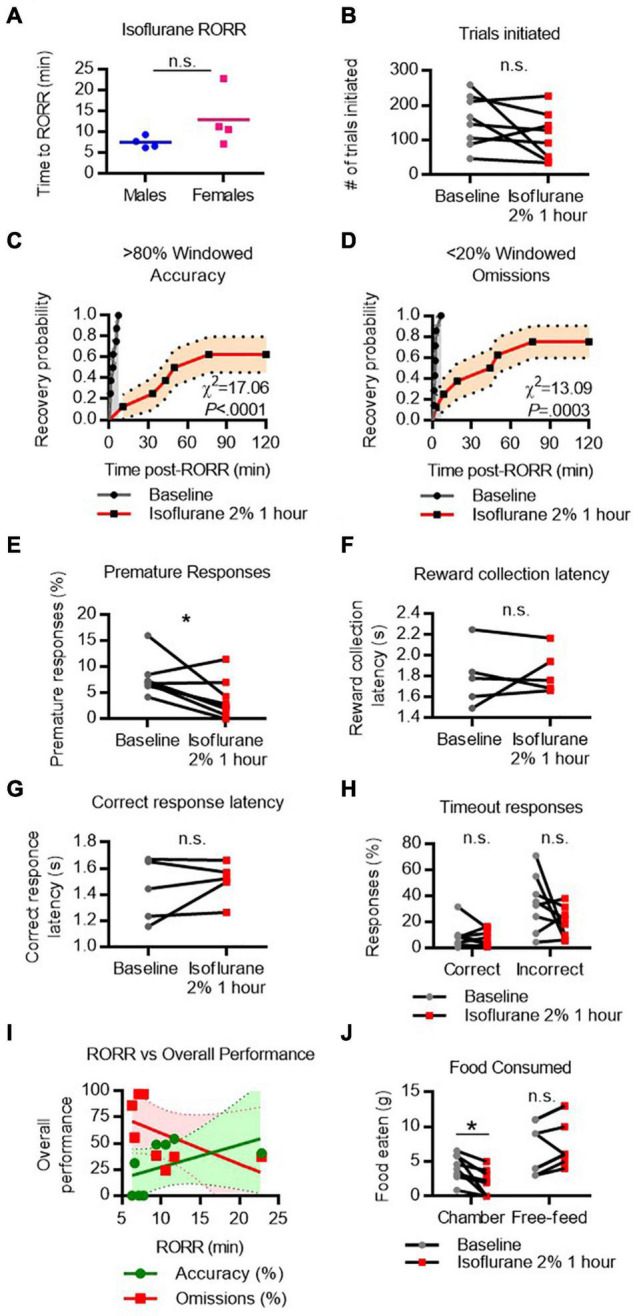
Performance on the 5-CSRTT following isoflurane. **(A)** Return of righting reflex (RORR) following 1 h of 2% isoflurane in male and female rats. **(B)** Total number of 5-CSRTT trials initiated at baseline and following isoflurane anesthesia. **(C,D)** Probability of achieving > 80% windowed accuracy **(C)** and < 20% windowed omissions **(D)** at baseline and following isoflurane by the indicated time. **(E–H)** Difference in premature responses **(E)**, reward collection latency **(F)**, correct response latency **(G)**, and timeout responses to correct and incorrect windows **(H)** at baseline and following isoflurane. **(I)** RORR latency vs. overall accuracy (green) and omission (red) performance following isoflurane anesthesia. Regression slope (solid line) and 95% confidence interval (dashed lines) indicated. **(J)** Total weight of food eaten in the chamber during the task and following the task during the free-feeding period at baseline and following isoflurane. n.s., not significant; *P < 0.05, *N* = 8.

To assess recovery of 5-CSRTT task performance after isoflurane, rats were placed in the testing chamber for 2 h following 1 h of 2% continuous isoflurane exposure. RORR latency following isoflurane was 7.5 ± 1.4 min in males and 13.0 ± 6.8 min in females (Figure 3A). To assess whether the ability to perform the task was affected by the isoflurane exposure, the total number of trial initiations following isoflurane was compared to the performance on the previous day (baseline). The number of trials initiated following RORR was not significantly different from baseline performance (Figure 3B), suggesting that the rats can engage with the task. Following RORR, 5 of 8 rats recovered windowed accuracy performance (Figure 3C) with a median recovery time of 63.1 min. Mantel-Cox log-rank comparison revealed that this was significantly delayed compared to baseline performance (χ^2^ = 17.06, *P* < 0.0001). In contrast, 6 of 8 rats achieved low windowed omission performance following RORR, with a median recovery time of 47.2 min (χ^2^ = 13.09, *P* = 0.0003 vs. baseline, Figure 3D). Following isoflurane, the number of premature responses decreased significantly from baseline [*t*(7) = 2.658, *P* = 0.0326, Figure 3E].

Because accuracy and omission rates, our primary outcome variables, could be a result of factors unrelated to higher-order cognitive processes, we assessed measures which relate to basic performance more broadly. Two measures associated with task motivation, reward collection latency (Figure 3F) and correct response latency (Figure 3G), were measured in the five animals that made at least one correct response following isoflurane exposure. Median reward collection latency and correct response latency were similar between baseline and following isoflurane, suggesting motivation to receive the food reward was not reduced following emergence.

We next compared the number of responses, as a percentage of total trials initiated, made to the correct and incorrect windows during the timeout period (Figure 3H). A large number of correct responses during the timeout period could indicate that rats are attending to the screen but are too slow to respond within the 5 s time window. However, RM-ANOVA revealed there was no main effect of isoflurane on time-out responses, suggesting omissions were not the result of impaired movement speed.

To determine whether rats that regained consciousness more quickly were more likely to perform better on the 5CSRTT, a regression analysis was performed on overall omission and accuracy performance against RORR latency (Figure 3I). RORR did not significantly predict overall omission nor accuracy performance on the 5-CSRTT.

Lastly, as the 5-CSRTT uses food as a reward, performance is affected by hunger levels. The weight of food eaten inside the chamber and during a 1 h free-feed period outside the chamber was compared at baseline and following isoflurane anesthesia (Figure 3J). Two-way RM-ANOVA comparing food location (during task vs. during free-feed) and drug condition (baseline vs. isoflurane) revealed a significant interaction [*F*_location__x__condition(1,__7)_ = 12.12, *P* = 0.0102]. Simple main effects revealed that following isoflurane rats eat significantly less food in the chamber [*t*(7) = 3.255, *P* = 0.0279], but will eat the same amount of food during the free-feeding period. These data indicate that poor performance on the task following isoflurane cannot be attributed to a lack of appetite, as rats will eat when given the opportunity.

### Cognitive Recovery Following Sevoflurane Emergence

**FIGURE 4:**
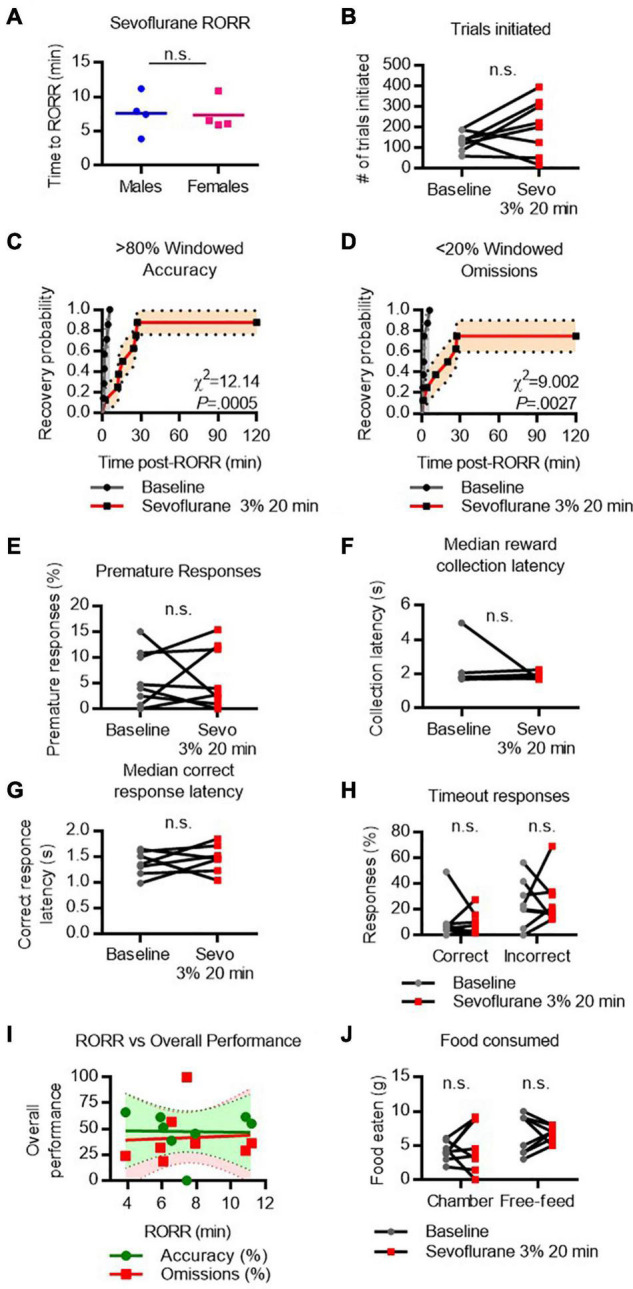
Performance on the 5-CSRTT following sevoflurane. **(A)** Return of righting reflex (RORR) following 20 min of 3% sevoflurane in male and female rats. **(B)** Total number of 5-CSRTT trials initiated at baseline and following sevoflurane anesthesia. **(C,D)** Probability of achieving > 80% windowed accuracy **(C)** and < 20% windowed omissions **(D)** at baseline and following sevoflurane by the indicated time. **(E,H)** Difference in premature responses **(E)**, reward collection latency **(F)**, correct response latency **(G)**, and timeout responses to correct and incorrect windows **(H)** at baseline and following sevoflurane. **(I)** RORR latency vs. overall accuracy (green) and omission (red) performance following sevoflurane anesthesia. Regression slope (solid line) and 95% confidence interval (dashed lines) indicated. **(J)** Total weight of food eaten in the chamber during the task and following the task during the free-feeding period at baseline and following sevoflurane. n.s., not significant; *N* = 8.

RORR latency following sevoflurane was 7.6 ± 3.0 min in males and 7.3 ± 2.4 min in females (Figure 4A). The total number of trials initiated following sevoflurane was comparable to baseline performance (Figure 4B). Following RORR, 7 of 8 rats achieved high windowed accuracy performance following sevoflurane anesthesia, with a median recovery time of 20.2 min which was significantly delayed compared to baseline (χ^2^ = 12.14, *P* = 0.0005, Figure 4C). In contrast, 6 of 8 rats achieved low windowed omission performance following RORR, with a median recovery time of 23.5 min (χ^2^ = 9.002, *P* = 0.0027 vs. baseline, Figure 4D). There was no statistical difference between the number of premature responses (Figure 4E), reward collection latency (Figure 4F), correct response latency (Figure 4G), nor timeout responses to correct or incorrect windows (Figure 4H). RORR latency was not predictive of overall accuracy nor omission performance on the 5-CSRTT (Figure 4I). Two-way RM-ANOVA revealed no main effect of sevoflurane on the amount of food eaten during or following the task (Figure 4J).

### Cognitive Recovery Following Propofol Emergence

**FIGURE 5:**
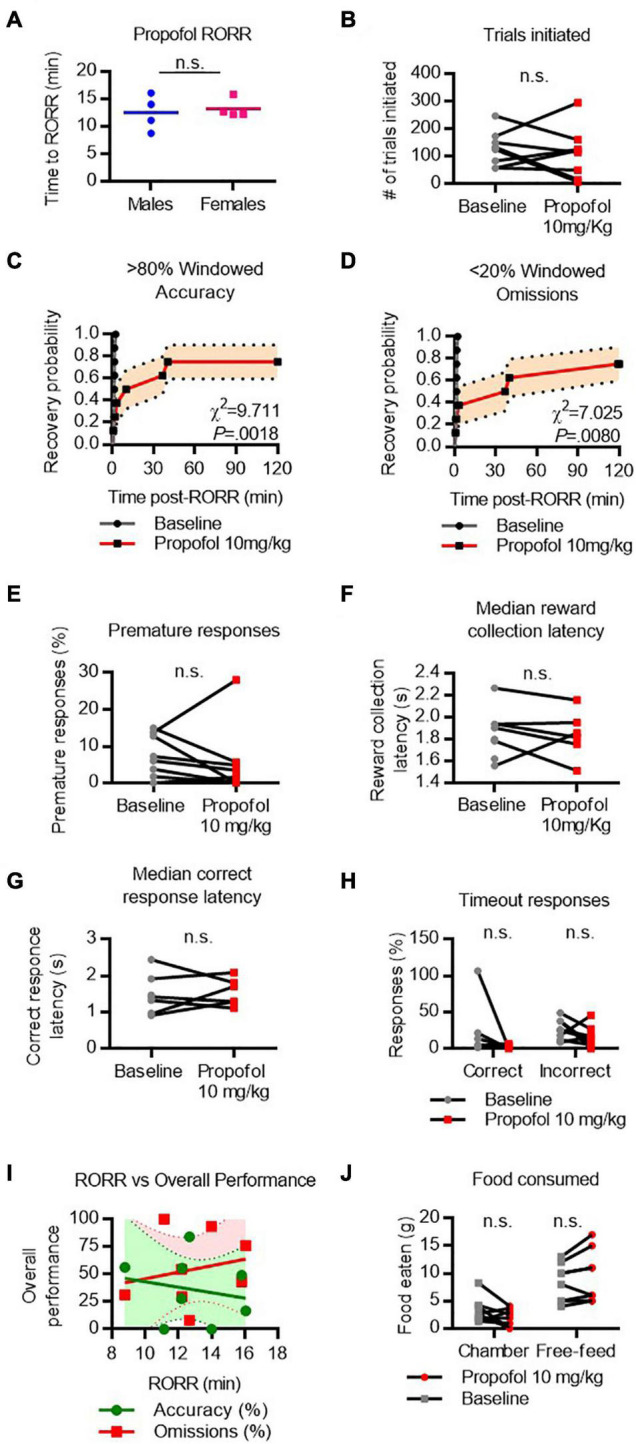
Performance on the 5-CSRTT following propofol. **(A)** Return of righting reflex (RORR) following a bolus dose of 10 mg/kg propofol I.V. in male and female rats. **(B)** Total number of 5-CSRTT trials initiated at baseline and following propofol anesthesia. **(C,D)** Probability of achieving > 80% windowed accuracy **(C)** and < 20% windowed omissions **(D)** at baseline and following propofol by the indicated time. **(E–H)** Difference in premature responses **(E)**, reward collection latency **(F)**, correct response latency **(G)**, and timeout responses to correct and incorrect windows **(H)** at baseline and following propofol. **(I)** RORR latency vs. overall accuracy (green) and omission (red) performance following propofol anesthesia. Regression slope (solid line) and 95% confidence interval (dashed lines) indicated. **(J)** Total weight of food eaten in the chamber during the task and following the task during the free-feeding period at baseline and following propofol. n.s., not significant; *N* = 8.

RORR following propofol (10 mg/kg I.V. bolus) was 12.5 ± 3.2 min in males and 13.23 ± 1.7 min in females (Figure 5A). The total number of trials completed did not differ between baseline and propofol anesthesia (Figure 5B). Following RORR, 7 of 8 rats achieved windowed accuracy performance following propofol anesthesia, with a median recovery time of 23.3 min (χ^2^ = 9.711, *P* = 0.0018 vs. baseline, Figure 5C). Six of 8 rats achieved low windowed omission performance following RORR, with a median recovery time of 38.5 min (χ^2^ = 7.025, *P* = 0.0080 vs. baseline, Figure 5D). There was no statistical difference between the number of premature responses (Figure 5E), reward collection latency (Figure 5F), correct response latency (Figure 5G), or timeout responses to correct or incorrect windows (Figure 5H). RORR latency was not predictive of overall accuracy nor omission performance on the 5-CSRTT (Figure 5I). Two-way ANOVA revealed no difference in the amount of food eaten between baseline and propofol conditions (Figure 5J).

### Cognitive Recovery Following Ketamine Emergence

**FIGURE 6:**
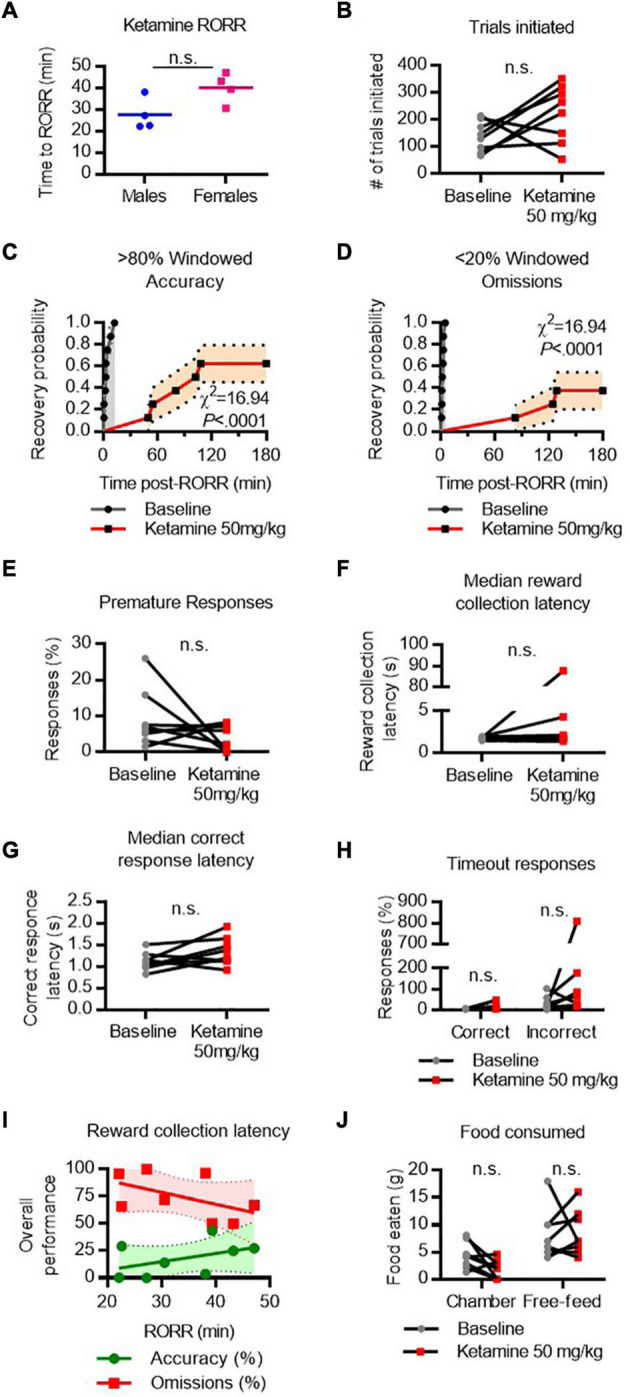
Performance on the 5-CSRTT following ketamine. **(A)** Return of righting reflex (RORR) following 50 mg/kg ketamine (infused over 10 min, I.V.) in male and female rats. **(B)** Total number of 5-CSRTT trials initiated at baseline and following ketamine anesthesia. **(C,D)** Probability of achieving > 80% windowed accuracy **(C)** and < 20% windowed omissions **(D)** at baseline and following ketamine by the indicated time. **(E–H)** Difference in premature responses **(E)**, reward collection latency **(F)**, correct response latency **(G)**, and timeout responses to correct and incorrect windows **(H)** at baseline and following ketamine. **(I)** RORR latency vs. overall accuracy (green) and omission (red) performance following ketamine. Regression slope (solid line) and 95% confidence interval (dashed lines) indicated. **(J)** Total weight of food eaten in the chamber during the task and following the task during the free-feeding period at baseline and following ketamine. n.s., not significant; *N* = 8.

RORR following ketamine (50 mg/kg I.V. infusion) was 27.55 ± 3.7 min in males and 40.05 ± 3.6 min in females (Figure 6A). The difference in RORR latencies was substantial between males and females, but not statistically significant [*t*(6) = 2.4, *P* = 0.0507]. The total number of trials initiated did not vary significantly between baseline and following ketamine (Figure 6B). Following RORR, 5 of 8 rats recovered accuracy performance, with a median recovery time of 104.9 min (χ^2^ = 16.94, *P* < 0.0001 vs. baseline, Figure 6C). However, only 3 of 8 rats recovered low omission performance (χ^2^ = 16.94, *P* < 0.0001 vs. baseline, Figure 6D) within the 3-h period given. There was no statistical difference between the number of premature responses (Figure 6E), reward collection latency (Figure 6F), nor correct response latency (Figure 6G).

Following ketamine, timeout response rates violated assumptions of normality, so Friedman’s test was performed to compare mean response rates. There was no significant difference in the percent of timeout responses to the correct or incorrect window (Figure 6H). RORR latency was not predictive of overall accuracy nor omission performance on the 5-CSRTT (Figure 6I). Two-way ANOVA revealed no difference in the amount of food eaten between baseline and ketamine conditions (Figure 6J).

### Cognitive Recovery Following Dexmedetomidine Emergence

To assess cognitive performance following dexmedetomidine, rats were initially dosed with 35 μg/kg which produced loss of righting in all rats; however, the poor performance on the task prompted the use of a second, lower dose (20 μg/kg). Notably, at this lower dose, rats were highly rousable in response to touch and could not be placed in a fully supine position without prompting a righting response. However, rats could be placed in a lateral recumbent position without causing immediate righting. Performance on the task following both doses was assessed.

**FIGURE 7:**
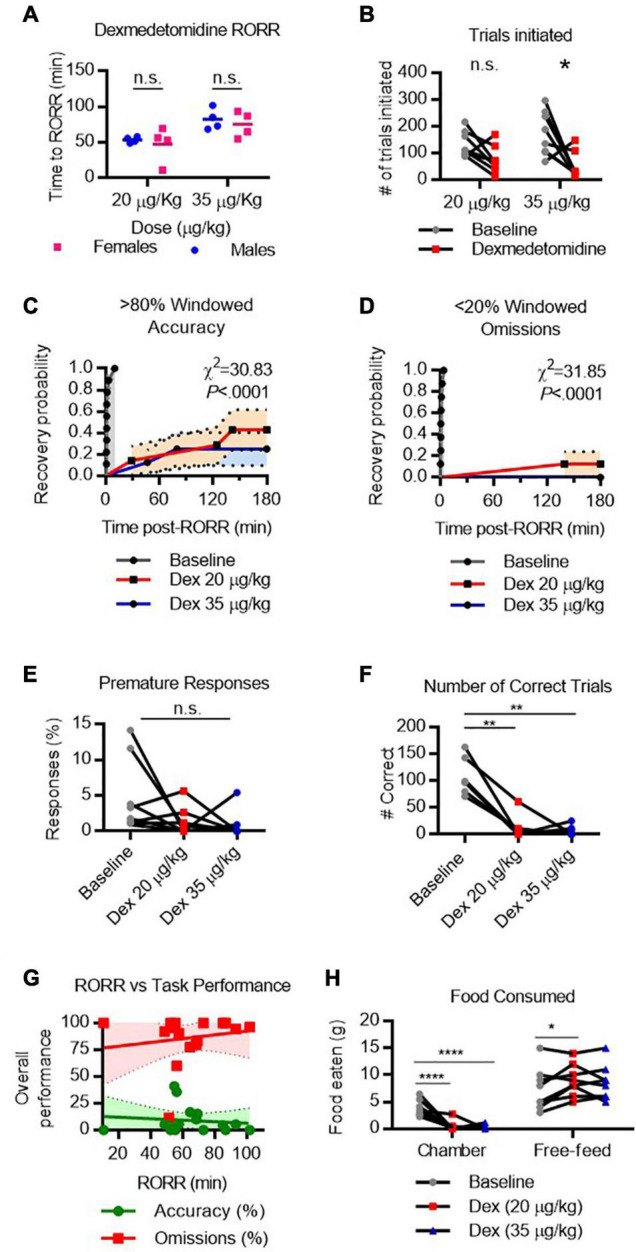
Performance on the 5-CSRTT following dexmedetomidine. **(A)** Return of righting reflex (RORR) following 20 μg/kg and 35 μg/kg dexmedetomidine (infused over 10 min, I.V.) in male and female rats. **(B)** Total number of 5-CSRTT trials initiated at baseline and following dexmedetomidine anesthesia. **(C,D)** Probability of achieving > 80% windowed accuracy **(C)** and < 20% windowed omissions **(D)** at baseline and following dexmedetomidine by the indicated time. **(E)** Percent of premature responses at baseline and following dexmedetomidine. **(F)** Total number of correct trials at baseline and following dexmedetomidine. **(G)** RORR latency vs. overall accuracy (green) and omission (red) performance following dexmedetomidine anesthesia. Regression slope (solid line) and 95% confidence interval (dashed lines) indicated. **(H)** Total weight of food eaten in the chamber during the task and following the task during the free-feeding period at baseline and following ketamine. n.s., not significant; *P < 0.05, **P < 0.01, ****P < 0.0001, *N* = 8.

RORR latency following dexmedetomidine varied by dose. Following 20 μg/kg IV (infused over 10 min), males recovered righting in 53.4 ± 4.0 min and females in 47.3 ± 25.3 min (Figure 7A). Following 35 μg/kg IV (infused over 10 min), males recovered righting in 82.2 ± 14.9 min and females in 75.0 ± 18.1 min. The two-way RM-ANOVA revealed a significant main effect of dexmedetomidine on the number of trials initiated [*F*_anesthetic(1,__7)_ = 23.03, *P* = 0.0020]. Dunnett’s *post hoc* comparison revealed that rats initiate significantly fewer trials following 35 μg/kg dexmedetomidine [*t*(7) = 3.049, *P* = 0.0372, Figure 7B] than at baseline. Following RORR, only 3 of 8 rats recovered windowed accuracy performance following 20 μg/kg dexmedetomidine, and only 2 of 8 rats recovered windowed accuracy performance following 35 μg/kg dexmedetomidine [χ_(2)_^2^ = 30.83, *P* < 0.0001, Figure 7C]. Only 1 of 8 rats regained low windowed omission performance following low-dose dexmedetomidine, and no rats regained low windowed omission performance following high-dose dexmedetomidine [χ_(2)_^2^ = 31.85, *P* < 0.0001, Figure 7D]. Premature responses dropped substantially following dexmedetomidine (Figure 7E). Because so few rats completed any correct trials (depicted in Figure 7F), reward collection latency and response latency could not be computed. RORR latency was not predictive of overall accuracy nor omission on the 5-CSRTT (Figure 7G).

Two-way RM-ANOVA revealed a significant interaction [*F*_location x condition__(2,__14)_ = 23.17, *P* < 0.0001] between anesthetic condition and location on the amount of food eaten. Dunnett’s test for *post hoc* comparisons was used to compare low-dose and high-dose dexmedetomidine conditions to baseline conditions. Unsurprisingly, poor task performance resulted in very little food eaten in the task chamber following either dose of dexmedetomidine (Figure 7H). However, during the free feeding period, rats ate more food following low-dose dexmedetomidine [*t*(14) = 2.746, *P* = 0.0289] than at baseline, suggesting they were compensating for missed eating opportunities during the testing. Overall, these results suggest that while rats do maintain an appetite following dexmedetomidine, their ability to perform the 5-CSRTT remains significantly impaired for hours following righting.

## Discussion

Here we describe a novel tool for assessing real-time cognitive recovery following general anesthesia in rats, the 5-CSRTT. Overall, we found that on average rats maintain stable performance throughout weeks of testing and make efforts to perform the task in the hours following anesthetic emergence. Importantly, young male and female rats developed no long-term cognitive impairment from repeated anesthetic exposures. Following isoflurane (2% for 1 h), sevoflurane (3% for 20 min), and propofol (10 mg/kg), most rats regain task competence within hours of righting. In contrast, following ketamine (50 mg/kg) or dexmedetomidine (20–35 μg/kg), the attentional demands of the task remained beyond reach for up to 3 h following righting. Interestingly, regardless of the anesthetic tested, the overall performance on the 5-CSRTT following anesthetic emergence was unrelated to individual differences in RORR latency. To our knowledge, these findings are the first to directly compare conscious recovery and cognitive recovery within a rodent model system.

The 5-CSRTT as a post-anesthetic testing paradigm was first introduced as an alternative to the spatial memory tasks typically used in animal models of postoperative delirium (Hambrecht-Wiedbusch et al., 2019). The study found that young, healthy rats maintain normal attentional performance on the 5-CSRTT between 1- and 7-days following isoflurane anesthesia, with or without ketamine. We similarly observed no change in the averaged baseline performance week-to-week following any of the anesthetic regimens tested, extending previous findings to a broader range of anesthetic agents. In addition, we adapted the task such that cognitive recovery could be monitored minute-to-minute following anesthetic emergence.

Our investigation revealed that most healthy, young rats are capable of organized, attentive, goal-oriented behavior in the hours following isoflurane, sevoflurane, and propofol emergence. It is important to emphasize that the young rats tested here have a low risk of developing postoperative cognitive complications. In humans, such complications are most common in the elderly or following emergency surgical procedures (Jin et al., 2020). Our results do deviate, however, from studies using learning and spatial memory tasks in rodents. Specifically, performance on the Morris Water Maze (Callaway et al., 2012) and the Radial Arm Maze (Culley et al., 2003, 2004a,2004b) has been shown to be negatively impacted by previous isoflurane exposure. In the study conducted by Callaway et al. (2012), 3- and 12-month-old rats were exposed to 1.2% isoflurane in 100% oxygen for 4 h and tested on the Morris Water Maze starting 1 week following anesthesia. While isoflurane exposure did not impair acquisition performance in either group, memory deficits were observed in the 3-month-old cohort (Callaway et al., 2012). In their first Radial Arm Maze study, Culley et al. (2003) found that 18-month-old rats display memory impairments in the radial arm maze 1–3 weeks following a 2-h exposure of 1.2% isoflurane/70% nitrous oxide, but 6-month-old rats show mild memory improvements. In a later study, the same group found that both 6- and 18-month-old rats display learning impairments in the days following the same anesthetic protocol (Culley et al., 2004b). The group later confirmed that learning impairments in aged rats are also observed when using 1.2% isoflurane in 100% oxygen (Culley et al., 2004a).

The differences between maze tasks and 5-CSRTT performance may in part be due to how these tasks assess cognitive function. Firstly, the 5-CSRTT requires that rats retrieve memories encoded prior to anesthesia—i.e., memory of the task rules—to engage with the task. In contrast, maze tasks assess how efficiently new memories are encoded following anesthesia. Secondly, unlike maze tasks, there is no new learning during the 5-CSRTT testing sessions. Instead, competence on the 5-CSRTT requires endogenous modulation of attentional control, spatial working memory, and inhibitory control in a familiar touchscreen-based testing environment (Robbins, 2002). In these respects, the 5-CSRTT closely reflects how cognitive assessments are conducted in clinical settings, particularly in the context of postoperative cognitive function (Lowery et al., 2008).

One notable result in our study was the severe task impairment following dexmedetomidine. In randomized control trials, dexmedetomidine has consistently been shown to reduce the prevalence of postoperative delirium when used in conjunction with other anesthetics (Fan et al., 2017). Dexmedetomidine is a highly selective α2-adrenergic receptor agonist (Virtanen et al., 1988). Its sedative effects are mediated by the reduction in norepinephrine release from the locus coeruleus, resulting in sedation without respiratory depression (Chiu et al., 1995; Scholz and Tonner, 2000). Hence, we were surprised to find that rats, despite being motivated to eat and highly rousable, would not engage with the task even 3 h after righting. However, these results are in line with the known involvement of the locus coeruleus on attention, arousal, and anesthetic emergence (Aston-Jones et al., 1999, 2000; Mansour et al., 2003; Bouret and Sara, 2004; Rowland and Kentros, 2008; Bast et al., 2018; Kelz et al., 2019). Indeed, chemogenetic inhibition of locus coeruleus noradrenergic neurons has been previously demonstrated to impair 5-CSRTT attentional performance in mice (Fitzpatrick et al., 2019). Further, in neural slice preparations, the inhibitory effect of dexmedetomidine on locus coeruleus neurons was only partially reversable after 2 h post-washout (Chiu et al., 1995). While it is unclear how these findings relate to physiologic conditions in the brain, they raise the possibility that protracted hypoactivity in the locus coeruleus following dexmedetomidine impairs attentional control in the 5-CSRTT, particularly when used as the sole intravenous anesthetic. These mechanisms warrant further exploration.

We also observed delayed recovery of omission performance following ketamine emergence. In both human (Ionescu et al., 2018) and rodent (Radford et al., 2018; Zhang et al., 2019, 2021) studies, even a single sub-anesthetic dose of ketamine has been shown to produce persistent changes in neural plasticity. Like dexmedetomidine, ketamine is typically administered in low doses as an adjunct with other general anesthetics. When administered intraoperatively, ketamine has been shown to attenuate postsurgical inflammation (Dale et al., 2012), and has been proposed to improve postoperative cognitive function. However, in a large, multi-center, randomized, double-blind control trial, intraoperative ketamine failed to reduce postoperative delirium, and was associated with a greater incidence of postsurgical hallucinatory events (Avidan et al., 2017). Interestingly, a more recent investigation of 626 surgical patients revealed that the risk of delirium in the postanesthetic care unit was four times greater when ketamine was used as an adjunct anesthetic (Hesse et al., 2019).

In the present study, we found that rats perform a substantial number of trials following ketamine, and most perform the task with a high degree of accuracy. Despite this, they were unable to continuously attend to the task, and subsequently missed a large number of the trials after initiating them. The confluence of these two results—high accuracy and high omissions—identified a persistent, altered state of consciousness unique to ketamine. These findings highlight the utility of the 5-CSRTT as a cognitive testing tool; rather than being an all-or-nothing test of cognitive function, deficits can manifest as specific performance impairments (Fitzpatrick et al., 2019).

While the 5-CSRTT confers several advantages in studying rodent cognitive function in the minutes and hours following anesthetic emergence, there are drawbacks to this technique. Primarily, training animals is time consuming and expensive. Starting with the first habituation session, animals took an average of 22 sessions to achieve a reliable, high level of performance. While more time- and cost-efficient methods for performing the 5-CSRTT have been proposed (Birtalan et al., 2020), the task is still far from being widely accessible. Another limitation of the 5-CSRTT technique is the requirement of food restriction. Hunger drives performance in operant conditioning tasks and, while we observed no loss of appetite in the present investigation, agents with anorexiant side effects may require special consideration when interpreting performance.

As a result of the substantial time and cost required to train animals in operant conditioning tasks, it is common practice to use the same animals to test multiple experimental conditions, as was done for the present investigation. Though average baseline performance was stable throughout testing, individual animals fluctuate week-to-week in their overall performance. In addition, though animals had a full week to recover between anesthetics, we cannot entirely discount order effects on cognitive recovery latency, particularly following drugs like ketamine which have known long-term neurocognitive effects. Another limitation in our study design is the small sample size, which makes detecting small effects sizes statistically difficult. It is also important to emphasize that the anesthetic regimens presented here cannot be directly compared to one another. Rather, this investigation only assesses recovery of cognitive function after RORR within each tested regimen.

Given these limitations, we still find that the 5-CSRTT offers a unique opportunity to investigate neurocognitive function in the post-anesthetic conscious state. Not only do operant conditioning tasks closely reflect human cognitive testing conditions, they also provide a clinically relevant endpoint for anesthetic recovery which cannot be predicted from measures of consciousness alone, such as RORR latency. Collectively, these results provide foundational data for which future investigations on anesthetic recovery may build.

## Data Availability Statement

The raw data supporting the conclusions of this article will be made available by the authors, without undue reservation.

## Ethics Statement

The animal study was reviewed and approved by the Massachusetts General Hospital Institutional Animal Care and Use Committee.

## Author Contributions

KV: conceptualization, methodology, investigation, data curation, and writing manuscript. EZ, RK, AC, and OM: investigation. KS: conceptualization, methodology, writing manuscript, and funding acquisition. All authors contributed to the article and approved the submitted version.

## Conflict of Interest

The authors declare that the research was conducted in the absence of any commercial or financial relationships that could be construed as a potential conflict of interest.

## Publisher’s Note

All claims expressed in this article are solely those of the authors and do not necessarily represent those of their affiliated organizations, or those of the publisher, the editors and the reviewers. Any product that may be evaluated in this article, or claim that may be made by its manufacturer, is not guaranteed or endorsed by the publisher.
